# Hepatic subcapsular hematoma post-ERCP: Case report and literature review

**DOI:** 10.1016/j.ijscr.2020.05.074

**Published:** 2020-06-06

**Authors:** Luca Giovanni Antonio Pivetta, Caroline Petersen da Costa Ferreira, João Paulo Venancio de Carvalho, Renata Yumi Lima Konichi, Victor Kenzo Fujikawa Kawamoto, Jose Cesar Assef, Mauricio Alves Ribeiro

**Affiliations:** a Emergency Service of the Irmandade da Santa Casa de São Paulo (ISCMSP), São Paulo, SP, Brazil; b Graduate in Surgery, Santa Casa de São Paulo School of Medical Sciences, São Paulo, SP, Brazil; c Graduate in Medicine, Santa Casa de São Paulo School of Medical Sciences, São Paulo, SP, Brazil

**Keywords:** Case report, Endoscopic retrograde cholangiopancreatography, Hepatic subcapsular hematoma, Subcapsular liver hematoma, Acute abdomen

## Abstract

**Introduction:**

Endoscopic retrograde cholangiopancreatography (ERCP) is one of the most frequently performed minimally invasive procedures currently available for diagnosis and treatment of biliary and pancreatic diseases. Though considered a safe procedure, it has the highest rate of complications among the other endoscopic procedures, such as duodenal perforation and hepatic subcapsular hematoma (HSH). We are a presenting a case report and review of the current literature.

**Method:**

We report one case HSH rupture, in a 25 years old female patient, 15 cm in diameter, affecting liver segments VI, VII and VIII, who underwent surgical treatment and performed a systematic literature review with the descriptors: endoscopic retrograde colangiopancreatography and hepatic subcapsular hematoma. All articles were reviewed and data on cases that presented rupture of the HSH analyzed separately.

**Results:**

Sixty one cases of HSH were described in the literature, fourteen of them ruptured.

When analyzing only the subgroup of patients who had ruptured subcapsular hematoma, we showed a significant increase in the mortality rate of patients when compared to non-ruptured (21.4% × 2.2%). We also report that patients with rupture required some type of intervention, of which 78.6% required surgery.

Conservative treatment may be the conduct and will suffice for most cases of non-ruptured hematomas. For patients who evolve with rupturing, surgical resolution, although non-mandatory, is necessary in most cases.

**Conclusion:**

HSH ruptured is a rare and potentially fatal post-ERCP complication whose treatment is eminently surgical.

## Highlights

• A case of hepatic subcapsular hematoma (HSH) rupture that required surgical treatment.
• Sixty one cases of HSH were described in the literature, fourteen of them ruptured.
• HSH rupture has a significant increase in the mortality (21.4% × 2.2%).
• Conservative treatment may be the conduct for cases with non-ruptured hematomas.
• HSH rupture required surgical intervention in 78.6% of cases.

## 1 Introduction

Endoscopic retrograde cholangiopancreatography (ERCP) is today one of the most commonly performed minimally invasive procedures for the diagnosis and treatment of biliary and pancreatic diseases. Although it is a safe method, ERCP has the highest incidence of complications among upper gastrointestinal endoscopic procedures [1,2], with complication rates ranging from 2.5%–8% when performed by experienced professionals [[3], [4], [5]].

Complications routinely described include acute pancreatitis (most common), acute cholangitis, hemorrhage, sepsis and cardiopulmonary changes [[6], [7], [8], [9], [10]]. However, there are some less common complications with high morbidity, such as duodenal perforation and hepatic subcapsular hematoma (HSH) [[11], [12], [13], [14]].

Regarding HSH, a extraluminal hemorrhagic complication with potential morbidity and mortality, there are 53 reports in literature, with a combined mortality rate of 7.5%, being 10 of those ruptured. The aim of this study is to perform a systematic review of HSH, an uncommon and high morbidity complication, and to report the case of a patient who presented with HSH after ERCP for choledocholithiasis treatment. The work has been reported in line with de SCARE criteria [15], and is approved by Santa Casa de São Paulo Research Ethics Committee number: 0897129.0.0000.5479.

## 2 Methods

A literature review was performed with the following descriptors: endoscopic retrograde cholangiopancreatography and hepatic subcapsular hematoma, in the following datebase: PubMed, scopus, BIREME.

Exclusion criteria was: articles that did not have at least an abstract available in one of the following languages: Italian, French, English and Portuguese. All articles were reviewed and data on cases with HSH rupture were analyzed separately.

The data was collected by two different authors, individually, and then analyzed, in case of any disagreement another author reviewed the original paper to minimize any error.

**Chart 1:**
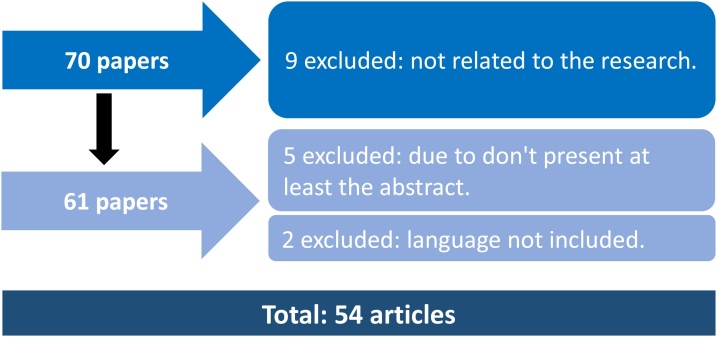
Flowchart: review process.

Of the initial 70 results, nine were excluded due to not being related to the research, 2 were excluded due to language and finally 5 were excluded due to not have at least an abstract available (Chart 1).

## 3 Case report

A 25-year-old female teacher, married, native to Argentina, with no comorbidities, BMI of 24, was admitted to with jaundice to the emergency department (Bilirubin 11.2 mg/dL), with secondary choledocholithiasis diagnosed by ultrasound (US) imaging. Abdominal US showed a normal-looking liver, dilated intra and extrahepatic biliary system, with 1.3 cm bile duct and 1.0 cm calculus inside, and ERCP was indicated for treatment. The procedure was successful, and calculus was removed with the help of guide wire and papillotomy, without complications. The gallbladder did not contrast during the exam.

**Fig. 1:**
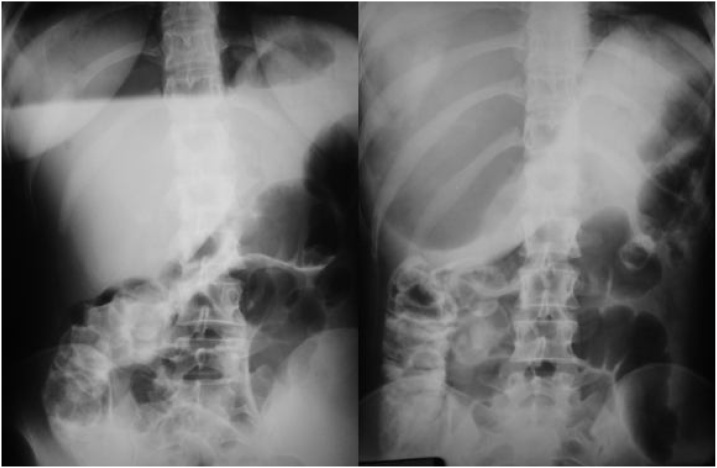
Abdominal x-ray showing gastric distension.

The patient returned to the ward hemodynamically stable and asymptomatic. After 8 h of ERCP, she experienced sudden abdominal pain and pallor, laboratory tests and abdominal radiography showed significant gastric distension (Fig. 1) and raised the hypothesis of blocked duodenal perforation, without pneumoperitoneum or complicated acute cholecystitis.

**Fig. 2:**
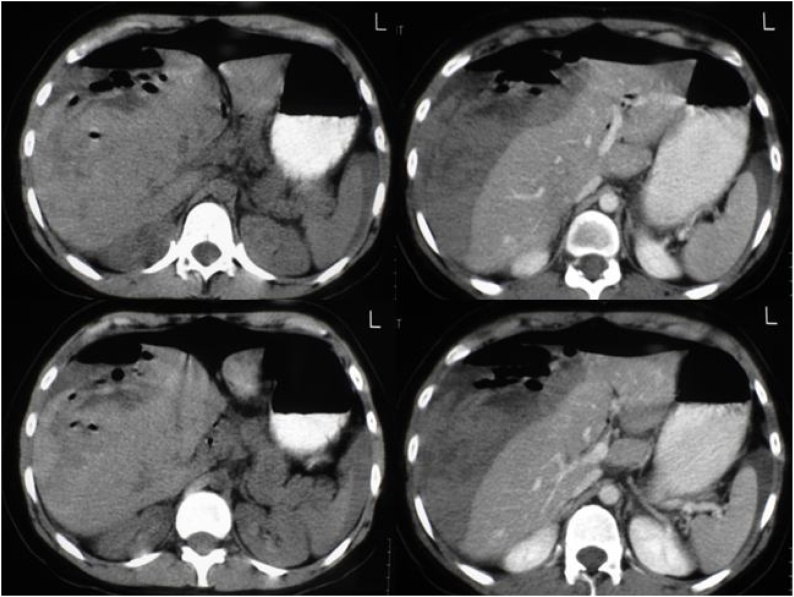
Abdominal CT showing HSH of about 15 cm, affecting liver segments 6, 7 and 8 with permeate air and small amount of perihepatic free liquid.

Antibiotic therapy was initiated with ciprofloxacin 400 mg every 12 h and metronidazole 500 mg every 8 h, and a CT scan of the abdomen was performed and showed subcapsular hematoma of about 15 cm in diameter, affecting liver segments VI, VII and VIII, with air and a small amount of perihepatic free fluid (Fig. 2). At this time, the patient presented with diffuse peritonitis on physical examination, and exploratory laparotomy was indicated.

**Fig. 3:**
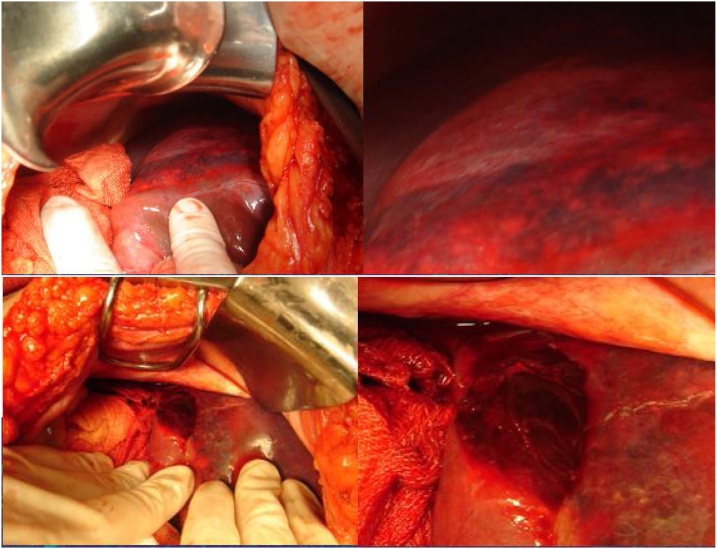
Intraoperative photography showing discrete hemoperitoneum, enlarged liver with HSH affecting right lobe with bleeding segment VI.

**Fig. 4:**
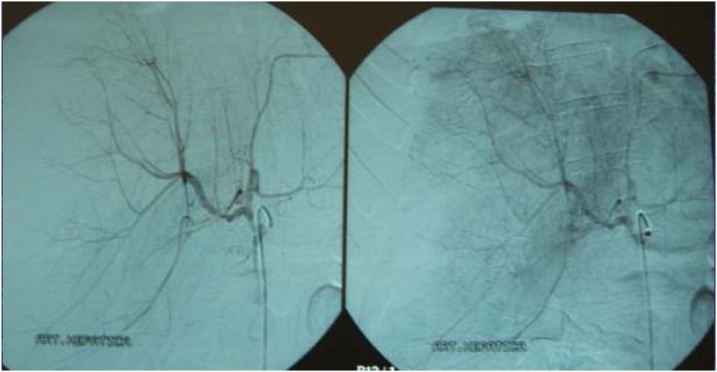
Arteriography with no signs of leakage.

Intraoperatively, a minimal amount of hemoperitoneum, a gallbladder with thick and delaminated walls, and subcapsular hematoma affecting the right lobe of the liver with oozing bleeding in segment VI were identified (Fig. 3). Patient was hemodynamically stable with 8.0 mg/dL hemoglobin (HB). Cholecystectomy and electrocautery hemostasis were performed in active bleeding, with apparent good final appearance, as well as a methylene blue test which ruled out duodenal perforation and cavity drainage. Postoperatively, after receiving 03 units of packed red blood cells, she maintained hemodynamic stability and was submitted to arteriography - in an attempt to identify the source of bleeding - with no signs of extravasation (Fig. 4).

On the fourth postoperative day (PO), she evolved with hemodynamic instability and 1500 mL of bloody outflow through the drain, and a new surgical approach was indicated. During the intraoperative period, moderate hemoperitoneum, subcapsular hematoma affecting the entire enlarged hepatic parenchyma, and hepatic bleeding in a laceration pattern were observed. Patient received 05 units of packed red blood cells and was submitted to argon-based hemostasis and tamponade with six compresses.

She remained intubated with ARDS (acute respiratory distress syndrome), maintaining a PaO2/Fio2 ratio of 60. Abdominal drainage was serosanguineous with an outflow rate of around 40 mL. After ventilatory improvement, a new laparotomy was performed, and compresses were removed. The liver was bruised on its entire surface, with no active bleeding, and enlarged (but smaller than in the previous approach). She was extubated on the 13th postoperative day and was discharged from the Intensive Care Unit (ICU) the following day, using antibiotics and pharmacologic venous thromboembolism prophylaxis.

**Fig. 5:**
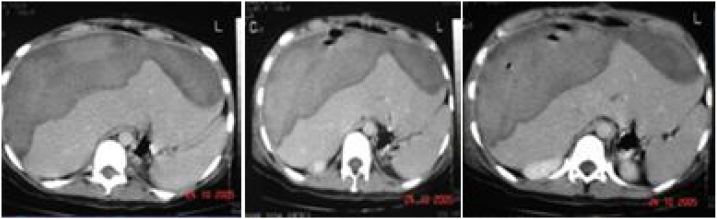
CT showing persistence of hepatic hematoma, no free liquid.

In the ward, the patient had some isolated fever peaks, with no defined source, and maintained antibiotic therapy with ciprofloxacin and metronidazole. On the 20th postoperative day, the patient was asymptomatic, with normal leukogram and sustained hemoglobin levels (Table 1). She underwent control a CT scan (Fig. 5) on the 22nd postoperative day, which still showed hepatic hematoma, without free fluid, and with no sings of thrombosis. The abdominal drain was removed, and the hospital discharge was scheduled to the following day. At night, the patient was asymptomatic in the ward when she experienced sudden dyspnea and died. At necropsy, the findings were pulmonary embolism and venous thrombosis in the pelvic plexus.

**Table 1:** Hemoglobin controls during hospitalization.

	OR	1° PO	4° PO	5° PO	13° PO	18° PO	22° PO
Hemoglobin (mg/dL)	13.8	7.9	5.0	10.0	9.6	10.2	10.1

## 4 Discussion

ERCP is an endoscopic procedure commonly performed since its introduction in 1968 [17] with established therapeutic and diagnostic utility. Its main indications are choledocholithiasis, biliary malignancies and benign or malignant pancreatic disorders [[17], [18], [19]].

The overall mortality rate of this procedure after diagnostic intervention is 0.2% [20] and after therapeutic procedure, 0.4–0.5% [14,[20,21] and with complication rates ranging from 2.5–8%, with pancreatitis being the most common complication (1–7%), followed by acute cholangitis (1.4%), hemorrhage (1%), duodenal perforation (<1%) [22] and cardiopulmonary complications (1%), such as arrhythmia, hypoxemia and aspiration [23]. Other less reported complications are: hepatic abscess formation, paralytic ileus, pneumothorax and pneumomediastinum [24,25], HSH, among others.

Hepatic subcapsular hematoma is an extraluminal hemorrhagic complication secondary to ERCP, whose pathology is poorly understood and potentially life-threatening, requiring early identification and treatment. HSH after ERCP is a rare complication, but may be more frequent than previously thought [14,26]. So far, 61 cases of HSH have been described in the literature, with 14 of them ruptured (Table 5).

The incidence of this complication may be underestimated, since most patients have no symptoms and post-ERCP monitoring is uncommon [27,28]. The first case was described in 2000 by Ortega et al. [29] and since then only a few isolated cases have been reported, including four (7.5%) death outcomes among them, demonstrating the potential severity of this condition.

The etiology of these hematomas is still unclear, and two hypotheses have been raised. The first one suggests that liver damage is secondary to the traction force exerted by the biliary duct extractor balloon when trying to remove a retained calculus. This force would cause the rupture of biliary vessels and branches, with subsequent bleeding [11,30]. The second hypothesis, more commonly reported in the literature, suggests that the guidewire – commonly used to cannulate the common bile duct – would perforate it [26,27,[31], [32], [33], [34]], thus damaging the juxtaposed hepatic parenchyma, causing rupture of small intrahepatic vessels. Blood filtration through the hepatic parenchyma, which follow a centrifugal pattern and the presence of a solid capsule would justify the presence of air in the hematoma and thus the pathophysiology. The high frequency of infection would be justified by the use of a guide wire without sterilization [35].

In the literature review conducted in this study, of the 61 cases analyzed, 49 reported the use of guidewire in the procedure (80.3%), while 12 of them made no mention of its use or not (19.7%).

Clinical manifestations of HSH are varied, including abdominal pain, shoulder pain, anemia, fever, and signs of hypotension. The literature review of this study showed that the most frequent clinical manifestation is abdominal pain (82.0%) followed by anemia (55.7%), hypotension (27.9%), fever (18.0%), and shoulder pain (13.1%) (Table 2) which corroborates the data obtained by Zizzo et al. [35] and Zappa [36].

**Table 2:** Sings and symptoms.

Signs and symptoms	
Abdominal pain	83.3% (50)
Anemia	56.7% (34)
Hypotension	28.3% (17)
Fever	18.3% (11)
Shoulder pain	13.3% (8)

The onset of these symptoms, associated with subsequent or immediate hypotension, is suggestive of the presence of HSH. Their manifestation may occur from hours to days after the procedure [5,37]. Our study revealed that 77.8% of clinical manifestations begin within 48 h (42 cases), 53.7% within 24 h (29 cases) and 40.7% within 12 h (22 cases), with a peak incidence 48 h after the procedure (12 cases, corresponding to 22.2%).

When analyzing the time of onset the HSH rupture cases shows a significant reduction in the median time 12 h (1–96 h) compared to 27 h (2 h – 15 days) in the HSH and average time, as in the average time 25.5 h HSH rupture group and 69,4 h HSH (Table 3).

**Table 3:** HSH x HSH rupture.

	HSH rupture	HSH
Cases	14 cases	45 cases
Average time before symptoms	25.5 h	69.4 h
Median time before symptoms	12 h (1–96 h)	27 h (2 h – 15 days)
Guidewire	12 (85.7%)	36 (80.0%)
Basket	1 (7.1%)	--
Mortality	3 (21.4%)	1 (2.2%)

The most predominant diagnostic methods for HSH are CT (91.4%) and US (22.4%). In the reported case, the patient experienced abdominal pain, the most frequently described symptom, and anemia 8 h after ERCP, with abdominal CT.

By analyzing only the subgroup of patients who had HSH rupture, we detected a significant increase in the mortality rate compared to non-ruptured (21.4% × 2.2%), even though the rupture subgroup was only 23.7% of the total number of cases reported in the literature. We also detected that patients with rupture required some type of intervention, of which 78.6% required surgery (Table 3).

The is a predominance HSH in right the lobe (87.3%) with 5.5% of the cases affecting both lobes; we also perform a statistic analyze, using Fisher's exact test, and no associations between death and the affected liver lobe side (p = 0.256) were verified.

Treatment should be customized on a case-by-case basis, but antibiotic therapy is always recommended due to the high risk of infection [12,26]. Hemodynamically stable patients with limited, non-compressive superficial hematoma can be managed conservatively [12]. Glisson’s capsule of the liver maintains hematoma stability and limits bleeding. In this approach, patient management consists of intravenous fluid infusion and replacement of blood derivatives, serial monitoring of hemoglobin concentration, serial hepatic function tests, repeated physical examination, rest and intensive care unit observation [3,26,29,38].

In addition, monitoring hematoma progression with imaging such as CT and US is the optimal approach. Whenever there is hemodynamic instability with active bleeding and contrast extravasation, immediate surgical or radiological approach should be considered [36].

Surgical treatment should be considered when patient’s general conditions becomes deteriorated, when there is hemodynamic instability, signs of peritoneal irritation, infected hematoma, findings of abdominal free fluid on CT [4] and complications, such as hematoma rupture [35,[39], [40], [41]]. In this case, the procedure consists of hematoma drainage, hemostasis with electrocauterization or hemostatic devices – if possible – and follow-up with imaging exams.

Should active bleeding and hemodynamic instability insue, it is reported in the literature that arteriography with bleeding source embolization has been used to control bleeding with satisfactory results [11,26,28,30]. Embolization by percutaneous angiography of a branch of the hepatic artery also proved to be an effective non-surgical treatment option [41].

A review of the literature revealed the predominance of conservative treatment (39.3%), followed by surgical approach (27.9%), percutaneous hematoma drainage (22.95%), and, finally, embolization treatment (8.2%) (Table 4).

**Table 4:** Treatment.

Treatment		
Surgery	17	27.9%
Conservative	24	39.3%
Percutaneous Drainage	14	22.95%
Percutaneous Drainage + Embolization	1	1.6%
Embolization	5	8.2%

**Table 5:** Review of the literature.

Author (Year)	ERCP Indication	Guidewire	Symptoms Onset Time	Sings and Syntoms	Rupture	Diagnosis Method	Hematoma Location	Treatment	Death
Ortega et al. [29] (2000)	Choledocolitiasis	Yes	—	Abdominal Pain	—	—	—	Percutaneous Drainage	No
Bhandarkar et al. [43] (2004)	Choledocolitiasis	Yes	10 days	Abdominal Pain, Anemia, Nausea and Pyrexia	No	Computed Tomography	Right Lobe (Segments V and VI)	Percutaneous Drainage	No
Chi et al. [26] (2004)	Pancreatic Neoplasm	Yes	5 h	Abdominal Pain and Anemia	Yes	Computed Tomography	Right Lobe	Embolization	No
Horn et al. [27] (2004)	Pancreatic Adenocarcinoma	Yes	48 h	Abdominal Pain and Anemia	No	Computed Tomography	—	Conservative	No
Ertugrul et al. [13] (2006)	Hilar Cholangiocarcinoma	Yes	48 h	Abdominal Pain, Anemia and Pyrexia	No	Computed Tomography and Ultrasonography	Right lobe (Segment V)	Conservative	No
Bhati et al. [34] (2007)	Choledocolitiasis	Yes	—	Abdominal Pain and Hypotension	Yes	Computed Tomography	Right Lobe	Percutaneous Drainage	No
Del Rossi et al. [44] (2007)	Choledocolitiasis	Yes	48 h	Abdominal Pain, Anemia and Hypotension	No	Computed Tomography and Ultrasonography	Right Lobe	Conservative	No
Papachristou et al. [45] (2007)	Hilar Cholangiocarcinoma	Yes	48 h	Abdominal Pain, Anemia and Shoulder Pain	—	Computed Tomography	Right Lobe	Conservative	—
Petit-Laurent et al. [46] (2007)	Choledocolitiasis	Yes	48 h	Abdominal Pain, Asthenia and Pyrexia	No	Computed Tomography and Ultrasonography	Right Lobe (Segment VIII)	Percutaneous Drainage	No
Priego et al. [41] (2007)	Choledocolitiasis	Yes	—	Abdominal Pain, Hypotension, Nausea, Shoulder Pain and Tachypnea	No	Computed Tomography	Right Lobe	Surgery	No
Cárdenas et al. [32] (2008)	Biliary Fistula after Liver Transplant	Yes	24 h	Abdominal Pain and Anemia	No	Computed Tomography and Ultrasonography	Left Lobe	Conservative	No
De La Serna - Higuera et al. [47] (2008)	Choledocolitiasis	Yes	48 h	Abdominal Pain and Leukocytosis	No	Computed Tomography and Ultrasonography	Right Lobe	Percutaneous Drainage	No
De Mayo et al. [48] (2008)	Ampullary Tumor	Yes	4 h	Shoulder Pain	No	Computed Tomography	Right Lobe	Conservative	No
McArthur et al. [33] (2008)	Choledocolitiasis	Yes	12 h	Abdominal Pain and Leukocytosis	No	Computed Tomography	Right Lobe	Conservative	No
Nari et al. [49] (2009)	Acute Biliary Pancreatitis	Yes	—	Abdominal Pain, Anemia, Nausea Pyrexia, Tachycardia, Tachypnea and Vomits	No	Computed Tomography and Ultrasonography	Right Lobe	Conservative	No
Yriberry Urena et al. [50] (2009)	Choledocolitiasis	Yes	48 h	Anemia	Yes	Computed Tomography	Right Lobe	Surgery	No
Revuelto Rey et al. [51] (2010)	Choledocolitiasis	Yes	6 h	Anemia	No	Computed Tomography	Right Lobe	Conservative	No
Saa et al. [52] (2010)	Choledocolitiasis	Yes	24 h	Hypotension and Upper Gastrointestinal Bleeding	No	Computed Tomography	Left Lobe	Surgery	Yes
Baudet et al. [30] (2011)	Choledocolitiasis	Yes	48 h	Abdominal Pain, Anemia, Hypotension and Pyrexia	Yes	Computed Tomography and Ultrasonography	Right Lobe (Segments VI, VII e VIII)	Embolization and Surgery	No
Del Pozo et al. [12] (2011)	Choledocolitiasis	Yes	5 days	Abdominal Pain and Anemia	No	Computed Tomography	Left Lobe and Right Lobe	Conservative	No
Manikam et al. [53] (2011)	Choledocolitiasis	Yes	14 h	Abdominal Pain, Pyrexia and Thoracic Pain	No	Computed Tomography	Right lobe (Segment VIII)	Percutaneous Drainage	No
Pérez - Legaz et al. [40] (2011)	Choledocolitiasis	Yes	2 h	Abdominal Pain, Anemia, Hypotension, Tachycardia and Tachypnea	Yes	Computed Tomography	Right Lobe (Segments V e VI)	Surgery with Electrocauterization	No
Shah et al. [54] (2011)	Benign Anastomotic Stenosis	Yes	—	—	No	—	—	Conservative	No
Weilert et al. [55] (2011)	Choledocolitiasis	Yes	24 h	Abdominal Pain	No	Computed Tomography	—	Conservative	No
Bartolo Rangel et al. [56] (2012)	Choledocolitiasis	—	24 h	Acute Abdomen and Shock	Yes	Thoracic Radiography and Intraoperative	—	Surgery	Yes
Orellana et al. [11] (2012)	Ampullary Tumor	—	4 h	Shoulder Pain	No	Computed Tomography	Right Lobe	Conservative	No
	Biliary Stent Exchange	—	2 h	Abdominal Pain, Hypotension and Tachycardia	Yes	Computed Tomography	Right Lobe	Embolization and Percutaneous Drainage	No
	Biliary Stent Exchange	—	—	Abdominal Pain and Shoulder Pain	No	Computed Tomography	Right Lobe	Conservative	No
Fei et al. [4] (2013)	Choledocolitiasis	Yes	2 h	Pyrexia	No	Computed Tomography	Right Lobe	Percutaneous Drainage	No
Klímová et al. [28] (2013)	Choledocolitiasis	Yes	6 h	Abdominal Pain and Anemia	No	Computed Tomography	Right Lobe	Embolization, Percutaneous Drainage and Surgery	No
Oliveira Ferreira et al. [57] (2013)	Choledocolitiasis	Yes	10 days	Abdominal Pain and Anemia	No	Computed Tomography and Ultrasonography	Right Lobe	Percutaneous Drainage	No
Patil et al. [58] (2013)	Choledocolitiasis	Yes	48 h	Abdominal Pain	No	Computed Tomography and Ultrasonography	Right Lobe	Percutaneous Drainage	No
Carrica et al. [59] (2014)	Choledocolitiasis	Yes	72 h	Abdominal Pain and Anemia	No	Magnetic Resonance Imaging and Ultrasonography	Right Lobe (Segments VII and VIII)	Percutaneous Drainage	No
Yoshii et al. [60] (2014)	Choledocolitiasis	—	30 h	Abdominal Pain	No	Computed Tomography	Right Lobe	Conservative	No
Gonzáles - López et al. [61] (2015)	Benign Choledoco Stenosis and Biliary Stent Exchange	Yes	72 h	Abdominal Pain, Anemia, Hypotension and Peritonitis	Yes	Computed Tomography	Right Lobe	Surgery	Yes
Zizzo et al. [35] (2015)	Choledocolitiasis	Yes	24 h	Abdominal Pain, Anemia, Hypotension and Shoulder Pain	No	Angiography and Computed Tomography	Right Lobe	Embolization	No
Curvale et al. [62] (2016)	Papilary Adenoma	Yes	1 h	Abdominal Pain, Anemia, Chills, Hypotension and Shoulder Pain	Yes	Computed Tomography	Right Lobe	Surgery	No
Ding Shi et al. [63] (2016)	Choledocolitiasis	Yes	16 h	Abdominal Pain and Anemia	No	Computed Tomography	Right Lobe	Conservative	No
Fiorini et al. [64] (2016)	Choledocolitiasis	Yes	8 h	Abdominal Pain and Pyrexia	No	Computed Tomography	Left Lobe (Segment II)	Percutaneous Drainage	No
Kilic et al. [65] (2016)	Choledocolitiasis	Yes	12 h	Abdominal Pain, Anemia, Fatigue and Hypotension	Yes	Computed Tomography and Ultrasonography	Left Lobe and Right Lobe	Surgery	No
Kisaoglu et al. [66] (2016)	Choledocolitiasis	Yes	2 h	Abdominal Pain and Right Pleural Effusion	No	Thoracic Computed Tomography	Right Lobe	Surgery	No
Servide et al. [37] (2016)	Choledocolitiasis	—	15 days	Abdominal Pain	No	Computed Tomography	Right Lobe (Segments IV e VIII)	Conservative	No
Solmaz et al. [67] (2016)	Choledocolitiasis	Yes	6 h	Abdominal Pain, Back Pain, Breathing Acidosis, Pyrexia and Shoulder Pain	No	Computed Tomography	Right Lobe	Conservative	No
Tamez et al. [68] (2016)	Choledocolitiasis	Yes	12 h	Abdominal Pain and Anemia	Yes	Ultrasound and Upper Digestive Endoscopy	Right Lobe	Surgery	No
Zappa et al. [36] (2016)	Choledocolitiasis	Yes	12 h	Abdominal Pain, Anemia, Hypotension and Tachycardia	No	Computed Tomography	Right Lobe (Segments VI e VII)	Embolization	No
Zela et al. [69] (2016)	Choledocolitiasis	—	10 days	Abdominal Pain	No	Ultrasonography	Right Lobe	Conservative	No
Corazza et al. [14] (2017)	Choledocolitiasis	Yes	2 h	Abdominal Pain and Anemia	No	Computed Tomography	Right Lobe (Segments IV, V, VI, VII and VIII)	Surgery	No
Del-Moral Martinez et al. [5] (2017)	Choledocolitiasis	—	6 h	Abdominal Pain, Anemia, Hypotension and Tachycardia	No	Computed Tomography	Left Lobe and Right Lobe	Conservative	No
	Choledocolitiasis	—	7 days	Abdominal Pain, Anemia and Pyrexia	No	Computed Tomography and Ultrasonography	Left Lobe (Segment III)	Percutaneous Drainage	No
De La Maza Ortiz et al. [70] (2018)	Choledocolitiasis	Yes	4 h	Anemia and Hypotension	No	Computed Tomography	Right Lobe	Conservative	No
	Choledocolitiasis	Yes	2 h	Abdominal Pain, Anemia and Hypotension	No	Angiography and Computed Tomography	Right Lobe	Embolization	No
Imperatore et al. [71] (2018)	Benign Biliary Stenosis and Biliary Stent Exchange	Yes	2 h	Abdomina Pain, Pyrexia and Thoracic Pain	Yes	Computed Tomography	Right Lobe	Surgery	No
	Choledocolitiasis	Yes	48 h	Abdominal Pain	No	Computed Tomography	Right Lobe	Embolization	No
Soler Humanes et al. [72] (2018)	Choledocolitiasis	—	—	Abdominal Pain and Anemia	No	Computed Tomography	Right Lobe	Percutaneous Drainage	No
Yang et al. [73] (2018)	Choledocolitiasis	Yes	96 h	Abdominal Distension, Abdominal Pain, Anemia and Pyrexia	Yes	Computed Tomography	Right Lobe	Percutaneous Drainage and Surgery	No
Lavall da Silva et al. [74] (2019)	Duodenal Papila Stenosis	—	9 days	Abdominal Pain, Anemia and Jaundice	No	Computed Tomography	Right Lobe (Segments V, VI, VII and VIII)	Conservative	No
Sommariva et al. [75] (2019)	Choledocolitiasis	Yes	48 h	Abdominal Pain and Anemia	No	Computed Tomography	Right Lobe (Segments VI, VII and VIII)	Conservative	No
Sotelo et al. [76] (2019)	Choledocolitiasis	Yes	96 h	Abdominal Pain and Dyspnea	No	Computed Tomography	Right Lobe	Percutaneous Drainage	No
Villavicencio et al. [77] (2019)	—	Yes	48 h	Abdominal Pain, Anemia and Hypotension	No	Computed Tomography	Right Lobe (Segments IV and VII)	Surgery	No
	—	—	15 days	—	No	—	—	Conservative	No
Case Report	Choledocolitiasis	Yes	8 h	Abdominal Pain and Anemia	Yes	CT	Right Lobe (Segments VI, VII and VIII)	Surgery	Yes

## 5 Conclusion

Hepatic subcapsular hematoma is a potentially fatal post-ERCP complication and should be considered as differential diagnosis of symptomatic post-procedure cases.

## Declaration of Competing Interest

No conflicts of interest relevant to this article.

## Sources of funding

At our own expenses.

## Ethical Approval

Ethics approval: Santa Casa de São Paulo Ethics and Research Committee in Reference number: **0897129.0.0000.5479**.

## Consent

Written informed consent was not obtained from the patient. The head of our medical team has taken responsibility that exhaustive attempts have been made to contact the family and that the paper has been sufficiently anonymised not to cause harm to the patient or their family. A copy of a signed document stating this is available for review by the Editor-in-Chief of this journal on request.

## Author contribution

Luca G A Pivetta, conceptualization, Validation, Writing - Original Draft ; Caroline P C Ferreira, Methodology, Validation ; João P V Carvalho, Formal analysis, Data Curation ; Renata Y L Konichi, Data Curation, Software, Writing - Review & Editing,; Victor K F Kawamoto, Data Curation, Software ; Jose C Assef, Writing - Review & Editing, Supervision ; Mauricio A Ribeiro, Methodology, Writing - Review & Editing, Supervision, Project administration.

## Registration of Research Studies

Case reports that are not first-in-man study already approved in Ethics Committee.

## Guarantor

Pivetta, L.G.A.

## Provenance and peer review

Not commissioned, externally peer-reviewed.
